# Gestational age as a predictor for subsequent preterm birth in New South Wales, Australia

**DOI:** 10.1186/s12884-021-04084-x

**Published:** 2021-09-06

**Authors:** Gavin Pereira, Annette K. Regan, Kingsley Wong, Gizachew A. Tessema

**Affiliations:** 1 Curtin School of Population Health, Curtin University, Perth, WA Australia; 2 Centre for Fertility and Health (CeFH), Norwegian Institute of Public Health, Oslo, Norway; 3 School of Nursing and Health Professions, University of San Francisco, San Francisco, USA; 4 Telethon Kids Institute, Nedlands, WA Australia

**Keywords:** Premature birth, Gestational age, Recurrence, Predictive value of tests, Sensitivity and specificity

## Abstract

**Background:**

There is no validated evidence base on predictive ability and absolute risk of preterm birth by gestational age of the previous pregnancy.

**Methods:**

We conducted a retrospective cohort study of mothers who gave birth to their first two children in New South Wales, 1994–2016 (*N* = 517,558 mothers). For each week of final gestational age of the first birth, we calculated relative and absolute risks of subsequent preterm birth.

**Results:**

For mothers whose first birth had a gestational age of 22 to 30 weeks the absolute risks of clinically significant preterm second birth (before 28, 32, and 34 weeks) were all less than 14%. For all gestational ages of the first child the median gestational ages of the second child were all at least 38 weeks. Sensitivity and positive predictive values were all below 30%.

**Conclusion:**

Previous gestational age alone is a poor predictor of subsequent risk of preterm birth.

## Introduction

Preterm birth, most commonly defined as birth before 37 weeks of completed gestation, has a global prevalence of approximately 11% of viable fetuses [1]. Because preterm birth can lead to insufficient time for organ development, it is associated with reduced fetal survival and substantially elevated risk of respiratory and neurodevelopmental morbidity among other sequelae [2]. The national prevalence of preterm birth in Australia is similar to the global average, at approximately 9%, but can vary considerably by ethnicity and obstetric history [3]. Previous preterm birth is a strong risk factor for preterm birth in later pregnancies [4–7]. Relative risks tend to be elevated at a similar gestational ages to the previous preterm birth [4]. Considerable attention has been directed toward the aetiology of recurrence [8], but less well-understood is how well final gestational age observed in the first pregnancy predicts final gestational age in the next pregnancy. Women are provided advice about modifiable risk factors when identified as being at risk of giving birth preterm based on the presence of previous preterm birth and other indicators [9] yet absolute risks of preterm birth based on previous pregnancy gestational age have not been comprehensively reported. We recently observed that although history of preterm birth is a strong risk factor for subsequent preterm birth, contrary to expectation, it was a poor predictor [10]. Inference from that study is limited because results have not yet been replicated in a large independent study population. To address this, we conducted a longitudinal study of all births in an independent cohort to estimate the absolute risk of preterm birth by final gestational age of the previous pregnancy, and to estimate predictive ability.

## Methods

### Study design and setting

This was a retrospective cohort study of births 1994–2016 in New South Wales (NSW), Australia.

### Participants and exclusions

Participants were mothers with two consecutive pregnancies at parity 0 and parity 1. From a starting population of 2,109,871 births, we sequentially excluded 62,511 multiple births; 602 births with missing final gestational age; 612 births with missing parity; 6382 births with erroneously recorded parity; and 3052 births with gestational ages < 22 weeks or > 44 weeks. The largest exclusion (*N* = 1,001,596 births) was attributable to restriction to the first two births (parity 0 and 1). After exclusions there were 517,558 mothers each with a parity 0 birth and parity 1 birth (*N* = 1,035,116 total births).

### Variables and data sources

Records of all stillborn and liveborn neonates in the state from the Perinatal Data Collection were obtained from the NSW Ministry of Health [11]. We used the clinical best estimate of final gestational age in *completed weeks*. A final gestational age of 34 weeks in completed weeks, for example, corresponds to birth at any obstetric day between *34 0/7* and *34 6/7* inclusive.

### Statistical analyses

We reported “absolute risk” of preterm birth and standard prediction metrics (sensitivity, specificity, and positive and negative predictive values) and “relative risks” to evaluate gestational age as a risk factor.

Gestational ages of the first child that were at the endpoints of the gestational age continuum were aggregated into categories: ≤ 30 weeks (*< 31 0/7*), 31–32 weeks (*31 0/7 to 32 6/7*), 42–44 weeks (*42 0/7 to 44 6/7*) [10]. At each week of final gestation of the first child we calculated three measures of risk of preterm birth of the second child: (i) relative risk of birth of the second child at < 28 weeks (*< 28 0/7*), < 32 weeks (*< 32 0/7*), < 34 weeks (*< 34 0/7*) and < 37 weeks (*< 37 0/7*) compared to mothers whose first child been born at 40 weeks (*40 0/7 to 40 6/7*) gestation; (ii) absolute risk of birth of the second child at < 28 weeks (*< 28 0/7)*, < 32 weeks (*< 32 0/7*), < 34 weeks (*< 34 0/7*) and < 37 weeks (*< 37 0/7*); and (iii) centiles (5th, 10th, 15th, 20th, 25th, 50th, 75th and 90th) of gestational age for the second child. Relative risk was calculated with log-binomial regression and 95% confidence intervals based on profile likelihoods for relative risks and Wilson score intervals for absolute risks [12, 13]. Relative risks were unadjusted to preserve the distribution of risk factors at each gestational age of the first child. All analyses were conducted with R v4.0.1 [14].

## Results

At first birth, 8.7% of mothers were of age < 20 years, 7.4% at age 35–39 years, and 0.5% at age ≥ 40 years (Table 1). The maternal age distribution among those who had preterm first births had relatively more younger and older mothers (10.6% of age < 20 years, 8.3% at age 35–39 years, and 0.8% at age ≥ 40 years). A small proportion (1.9%) of the population were of Aboriginal ethnicity but a larger proportion of (3.0%) of preterm first births were to Aboriginal women. The prevalence of smoking during pregnancy for the first birth was 12.2% for the whole cohort and 15.9% for the mothers that whose first birth was preterm. The socioeconomic disadvantage index for the study population (mean of 997) was similar to the national population (mean of 1000) but less variable (standard deviation of 66 compared to 100). The socioeconomic disadvantage index for mothers whose first birth was preterm was lower (mean of 993) than that for the whole cohort and less variable (standard deviation of 65). The prevalance of stillbirth when the first birth was preterm (6.6%) was more than ten times larger than for the cohort as a whole (0.6%). In contrast, the proxy for fetal growth restriction (small for gestational age) was less prevalent for the first preterm births (11.8%) than for the whole cohort (12.2%).

**Table 1:** Characteristics at the first birth of the study population of mothers who gave birth to their first two singleton children between 1994 and 2016 in New South Wales (*N* = 517,558 mothers)

	All	Term	Preterm
	N (%)	N (%)	N (%)
All	517,558 (100)	487,041 (94.1)	30,517 (5.9)
Maternal Age			
< 20 years	44,818 (8.7)	41,588 (8.5)	3230 (10.6)
20–24 years	111,386 (21.5)	104,945 (21.5)	6441 (21.1)
25–29 years	181,503 (35.1)	171,489 (35.2)	10,014 (32.8)
30–34 years	138,676 (26.8)	130,624 (26.8)	8052 (26.4)
35–39 years	38,307 (7.4)	35,780 (7.3)	2527 (8.3)
≥ 40 years	2737 (0.5)	2492 (0.5)	245 (0.8)
Missing	131 (< 0.1)	123 (< 0.1)	8 (< 0.1)
Ethnicity			
Aboriginal or Torres Strait Islander ^a^	9883 (1.9)	8982 (1.8)	901 (3.0)
Non-Aboriginal	410,248 (79.3)	386,295 (79.3)	23,953 (78.5)
Missing	97,427 (18.8)	91,764 (18.8)	5663 (18.6)
Smoked during pregnancy ^b^			
No	453,201 (87.6)	427,638 (87.8)	25,563 (83.8)
Yes	63,284 (12.2)	58,419 (12.0)	4865 (15.9)
Missing	1073 (0.2)	984 (0.2)	89 (0.3)
Year of birth			
1994–1999	145,603 (28.1)	137,052 (28.1)	8551 (28.0)
2000–2004	124,706 (24.1)	117,300 (24.1)	7406 (24.3)
2005–2009	134,350 (26.0)	126,505 (26.0)	7845 (25.7)
2010–2016	112,899 (21.8)	106,184 (21.8)	6715 (22.0)
Small for gestational age			
No	453,865 (87.7)	427,054 (87.7)	26,811 (87.9)
Yes	63,214 (12.2)	59,616 (12.2)	3598 (11.8)
Missing	479 (0.1)	371 (0.1)	108 (0.4)
Stillbirth			
No	514,568 (99.4)	486,070 (99.8)	28,498 (93.4)
Yes	2990 (0.6)	971 (0.2)	2019 (6.6)
Socioeconomic Index ^c^			
Missing	4103 (0.8)	3880 (0.8)	223 (0.7)
Mean (SD) ^d^	997 (66)	997 (66)	993 (65)

^a^Aboriginal or Torres Strait Islander

^b^Smoked during pregnancy

^c^Index of Relative Socioeconomic Disadvantage (IRSD) at Statistical Local Area level

^d^*SD* Standard deviation. The national mean and standard deviation of the IRSD index are 1000 and 100 respectively

For mothers whose first birth had a gestational age of 22 to 30 weeks the relative risk of second birth before 28, 32, and 34 weeks’ gestation was between 14 and 21 times higher; and the relative risk of second birth before 37 weeks was less than 12 times higher than those whose first birth was at 40 weeks’ gestation. Absolute risk of the second child birth before 28 weeks’ and 30 weeks’ gestation corresponding to all first child gestational ages were less than 5 and 10%, respectively (Table 2). The upper interval estimate limits of absolute risk of second child birth before 34 weeks’ were less than 11%.

**Table 2:** Relative and absolute risk^a^ of preterm birth of the second child^b^ by gestational age of the first child for the study population for mothers who gave birth to their first two singleton children between 1994 and 2016 in New South Wales (*N* = 517,558 mothers)

First Child		Preterm Birth of Second Child				Preterm Birth of Second Child				Gestational Age of Second Child							
		< 28 weeks	< 32 weeks	< 34 weeks	< 37 weeks	< 28 weeks	< 32 weeks	< 34 weeks	< 37 weeks								
	N (%)	1629 (0.31)	3688 (0.71)	6276 (1.21)	23,940 (4.63)	1629 (0.31)	3688 (0.71)	6276 (1.21)	23,940 (4.63)	Centile of Distribution							
Gest. Age (weeks)		Relative Risk (95% Confidence Interval ^c^)				Absolute Risk (%, 95 Confidence Interval ^d^)				5	10	15	20	25	50	75	90
22–30	4371 (0.8)	16.65 (13.83–19.95)	18.38 (16.27–20.71)	16.88 (15.34–18.54)	10.37 (9.79–10.96)	3.66 (3.14–4.26)	8.42 (7.63–9.28)	12.77 (11.81–13.79)	27.45 (26.15–28.80)	29	32	34	35	36	38	39	40
31–32	2338 (0.5)	7.59 (5.38–10.38)	11.95 (9.91–14.28)	12.55 (10.92–14.35)	10.27 (9.54–11.03)	1.67 (1.22–2.27)	5.47 (4.62–6.47)	9.50 (8.37–10.75)	27.20 (25.44–29.04)	31	34	35	36	36	38	39	40
33	2063 (0.4)	6.61 (4.47–9.39)	8.99 (7.17–11.13)	10.38 (8.83–12.11)	10.52 (9.75–11.33)	1.45 (1.02–2.07)	4.12 (3.34–5.07)	7.85 (6.77–9.09)	27.87 (25.98–29.85)	32	34	35	36	36	38	39	40
34	3569 (0.7)	4.59 (3.21–6.35)	6.42 (5.22–7.81)	7.52 (6.49–8.66)	8.20 (7.65–8.78)	1.01 (0.73–1.39)	2.94 (2.44–3.55)	5.69 (4.97–6.50)	21.71 (20.39–23.10)	33	35	36	36	37	38	39	40
35	6062 (1.2)	4.35 (3.27–5.68)	4.75 (3.94–5.68)	5.93 (5.21–6.73)	7.06 (6.64–7.49)	0.96 (0.74–1.23)	2.18 (1.84–2.58)	4.49 (3.99–5.04)	18.69 (17.73–19.69)	34	35	36	37	37	38	39	40
36	12,114 (2.3)	2.55 (1.96–3.28)	3.32 (2.82–3.88)	3.80 (3.37–4.26)	5.35 (5.08–5.64)	0.56 (0.44–0.71)	1.52 (1.32–1.75)	2.87 (2.59–3.19)	14.18 (13.57–14.81)	35	36	37	37	37	38	39	40
37	26,310 (5.1)	2.39 (1.96–2.89)	2.61 (2.29–2.97)	2.73 (2.47–3.01)	3.70 (3.53–3.87)	0.52 (0.44–0.62)	1.20 (1.07–1.34)	2.06 (1.90–2.24)	9.79 (9.44–10.16)	35	37	37	37	38	39	39	40
38	68,508 (13.2)	1.31 (1.10–1.56)	1.61 (1.44–1.80)	1.79 (1.64–1.95)	2.29 (2.20–2.39)	0.29 (0.25–0.33)	0.74 (0.68–0.80)	1.35 (1.27–1.44)	6.08 (5.90–6.26)	36	37	38	38	38	39	40	40
39	115,699 (22.4)	1.20 (1.03–1.40)	1.22 (1.10–1.36)	1.24 (1.14–1.34)	1.46 (1.40–1.52)	0.26 (0.24–0.30)	0.56 (0.52–0.61)	0.94 (0.88–1.00)	3.86 (3.75–3.98)	37	38	38	38	38	39	40	40
40	168,287 (32.5)	1	1	1	1	0.22 (0.20–0.24)	0.46 (0.43–0.49)	0.76 (0.72–0.80)	2.65 (2.57–2.73)	37	38	38	38	39	39	40	41
41	96,443 (18.6)	0.94 (0.79–1.12)	0.89 (0.79–1.00)	0.83 (0.75–0.91)	0.78 (0.74–0.82)	0.21 (0.18–0.24)	0.41 (0.37–0.45)	0.63 (0.58–0.68)	2.07 (1.98–2.16)	38	38	38	39	39	40	40	41
42–44	11,794 (2.3)	1.00 (0.66–1.46)	1.00 (0.75–1.30)	0.87 (0.69–1.09)	0.77 (0.67–0.87)	0.22 (0.15–0.32)	0.46 (0.35–0.60)	0.66 (0.53–0.82)	2.03 (1.79–2.30)	38	38	38	39	39	40	41	41

^a^Results reported to two decimal places, which is accurate when first child was not preterm (birth from 37 weeks). The results are accurate to 1 decimal place if the first child was preterm (birth before 37 weeks)

^b^< 28 weeks (< 28 0/7), < 32 weeks (< 32 0/7), < 34 weeks (< 34 0/7), < 37 weeks (< 37 0/7)

^c^Profile likelihood confidence interval

^d^Wilson score confidence interval

### Distribution of second child gestational age by first child gestational age

The median second child gestational age remained at or above 38 weeks irrespective of the gestational age in the previous birth. The lowest quartile of second child gestational age was at least 36 weeks (Table 2). The lowest decile (10th decile) of second child gestational age remained below 34 weeks if the gestational age of the first child was 22–30 weeks, and was at least 34 weeks otherwise.

### Ability of first child preterm birth to predict second child preterm birth

First child preterm birth before 37 weeks’ gestation achieved the highest sensitivity for predicting second child preterm birth prior to 28, 32, 34 and 37 weeks’ gestation (Table 3). However, for all categorisations of preterm birth, fewer than 26% of mothers with a second child born preterm had a first child born preterm. Moreover, fewer than 29% of mothers with a first child born preterm had a second child born preterm, for all categorisations of preterm birth.

**Table 3:** Ability^a^ of preterm birth^b^ of the first child to predict preterm birth of the second child for the study population for mothers who gave birth to their first two singleton children between 1994 and 2016 in New South Wales (*N* = 517,558 mothers)

		Preterm Birth of Second Child							
		Sensitivity (%, 95 Confidence Interval ^c^)				Positive Predictive Value (%, 95 Confidence Interval ^c^)			
		< 28 weeks	< 32 weeks	< 34 weeks	< 37 weeks	< 28 weeks	< 32 weeks	< 34 weeks	< 37 weeks
Preterm Birth of First Child	< 28 weeks	6.57 (5.46–7.88)	5.64 (4.94–6.43)	4.76 (4.26–5.32)	2.60 (2.40–2.81)	4.35 (3.62–5.24)	8.47 (7.43–9.63)	12.17 (10.94–13.52)	25.32 (23.64–27.07)
	< 32 weeks	10.62 (9.22–12.21)	11.31 (10.32–12.37)	10.33 (9.60–11.10)	6.04 (5.75–6.35)	3.26 (2.82–3.78)	7.87 (7.17–8.63)	12.23 (11.37–13.14)	27.29 (26.11–28.50)
	< 34 weeks	14.06 (12.45–15.83)	15.75 (14.61–16.97)	15.01 (14.15–15.91)	10.07 (9.70–10.46)	2.61 (2.30–2.97)	6.62 (6.12–7.16)	10.74 (10.11–11.40)	27.49 (26.56–28.43)
	< 37 weeks	24.00 (21.99–26.14)	27.17 (25.76–28.63)	28.12 (27.02–29.25)	25.22 (24.67–25.77)	1.28 (1.16–1.41)	3.28 (3.09–3.49)	5.78 (5.53–6.05)	19.78 (19.34–20.23)
		Specificity (%, 95 Confidence Interval ^c^)				Negative Predictive Value (%, 95 Confidence Interval ^c^)			
		< 28 weeks	< 32 weeks	< 34 weeks	< 37 weeks	< 28 weeks	< 32 weeks	< 34 weeks	< 37 weeks
	< 28 weeks	99.54 (99.53–99.56)	99.56 (99.54–99.58)	99.58 (99.56–99.60)	99.63 (99.61–99.64)	99.70 (99.69–99.72)	99.32 (99.30–99.35)	98.84 (98.81–98.87)	95.47 (95.42–95.53)
	< 32 weeks	99.01 (98.98–99.03)	99.05 (99.02–99.08)	99.09 (99.06–99.12)	99.22 (99.19–99.24)	99.72 (99.70–99.73)	99.36 (99.34–99.38)	98.90 (98.87–98.93)	95.61 (95.55–95.66)
	< 34 weeks	98.34 (98.31–98.38)	98.41 (98.37–98.44)	98.47 (98.43–98.50)	98.71 (98.68–98.74)	99.72 (99.71–99.74)	99.39 (99.37–99.41)	98.95 (98.92–98.98)	95.77 (95.71–95.82)
	< 37 weeks	94.16 (94.10–94.22)	94.26 (94.19–94.32)	94.38 (94.31–94.44)	95.04 (94.98–95.10)	99.75 (99.73–99.76)	99.45 (99.43–99.47)	99.07 (99.05–99.10)	96.32 (96.27–96.38)

^a^Sensitivity: Proportion of second preterm births for which the first birth was preterm; Positive Predictive Value: Proportion of first preterm births for which the second birth was preterm; Specificity: Proportion of second births that were not preterm for which the first birth was also not preterm; Negative Predictive Value: The proportion of first births that were not preterm for which the second birth was also not preterm

^b^Preterm: < 28 weeks, < 32 weeks, < 34 weeks, < 37 weeks

^c^Wilson score confidence interval

### Trend in preterm birth of firstborn children during the study period

For firstborn preterm birth before 28 weeks we observed an increase of 11% every 5 years (RR 1.11, 95% CI: 1.07–1.14). The other preterm birth classifications had a stable trend.

## Discussion

The absolute risks, sensitivity and positive predictive values observed for this cohort imply that preterm birth of a woman’s first child, overall and when categorised were poor predictors for preterm birth of the next, pregnancy with the next child. Our findings validate conclusions for the study from Western Australia [10]. The median gestational age of second births was 38 weeks (or more) irrespective of the gestational age of the first child, and even for the mothers whose first children were extremely preterm (< 28 weeks), the absolute risk of clinically significant preterm birth (< 34 weeks) was less than 14%. The findings in our study are reassuring for couples planning another pregnancy if their first child was preterm. Summaries of absolute risk of second child preterm birth by first child gestational age (week) is useful for communication, most notably postpartum family planning after experiencing preterm birth.

Our results were derived from a large Australian cohort (NSW) of singleton births for which the prevalence of preterm birth in first pregnancy was 5.9%. Point and interval estimates were largely consistent with the results from our previous study [10]. The slightly higher absolute risks observed in the previous study might be attributed to the contribution of births early in the study period, which ranged from 1980 to 2015 in that study (cf 1994 to 2016 in the present study) or slightly more higher risk pregnancies in WA than NSW. Compared to NSW, WA has a higher proportion of the population from rural and remote communities (16% vs 7%), from Aboriginal backgrounds (3% vs 2%), and less total health expenditure (AUD 3905 vs AUD 3970 per capita) [15].

Screening whole populations based on attributes of the previous pregnancy such as gestational age [16–19] would usually necessitate high predictive performance, which we have now confirmed in two independent populations cannot be achieved using previous gestational age alone but might achieve better risk stratification if used with other information [20]. A limitation of this study is that it relied on data already collected through routine perinatal registrations and consequently we could not estimate absolute risk of preterm birth by previous gestational age separately in the presence and absence of treatment. Treatments used to prolong pregnancy inherently decrease risk of preterm birth. Treatments such as tocoloytic treatments, which temporarily delay preterm labour, are not likely to influence our study results because the treatment decision is not conditional on the gestational age of the previous pregnancy. Treatments, such as progesterone prophylaxis, that are recommended for women based on previous preterm birth experience would decrease the risk of preterm birth. Because we did not have information on treatment, our study provides absolute risk estimates that are averaged across all treatment conditions. Knowledge of absolute risk by treatment scenario would be useful during post-partum counselling, particularly for families who plan to have a child but would otherwise decline treatment.

We posit that the first step towards both elucidating the aetiology of recurrence as well as the development of individualised prediction models is the production of a reference for preterm birth or gestational age, based on previous gestational age. Notable regions for replication include Japan, for which national recurrence risk estimates were recently reported by Seyama et al. [21]; regions described in the review on recurrence undertaken by Kazemier et al. [22]; and countries with well-established probabilistic or deterministic data linkage systems that enable longitudinal linkage, such as those in the Nordic countries [23].

## Conclusion

Our results confirm that gestational age is strongly associated with subsequent preterm birth, but is a poor predictor of subsequent preterm birth, and provides an evidence-base for communicating the absolute risks during post-partum counselling. This evidence-base might be used to either identify higher-risk mothers for counselling regarding modifying other risk factors, but also serves to minimise unintentional misinformation which overstates risk among mothers who have experienced a preterm birth and want to know their risk of experiencing a subsequent preterm birth.

## Data Availability

The data underlying this study are not publicly available due to a data use agreement that restricts our ability to share the unit record data on ethical and legal grounds. The data are sensitive, and de-identified but potentially re-identifiable. Requests to access perinatal registrations data can be submitted to the Ministry of Health, New South Wales, Australia, with application and contact information located here: https://www.cherel.org.au/apply-for-linked-data.
